# “We’ve got through hard times before” : acute mental distress and coping among disadvantaged groups during COVID-19 lockdown in North India - a qualitative study

**DOI:** 10.1186/s12939-020-01345-7

**Published:** 2020-12-17

**Authors:** Kaaren Mathias, Meenal Rawat, Sharad Philip, Nathan Grills

**Affiliations:** 1 Burans, Herbertpur Christian Hospital, Attenbagh, Herbertpur, Uttarakhand India; 2 Psychiatric Rehabilitation Department, NIMHANS, Bengaluru, India; 3 Nossal Institute, University of Melbourne and Australia- India Institute, Melbourne, Australia

**Keywords:** COVID-19, Mental health, Qualitative, Disability, India, Equity

## Abstract

**Background:**

The COVID-19 crisis in India negatively impacted mental health due to both the disease and the harsh lockdown, yet there are almost no qualitative studies describing mental health impacts or the strategies of resilience used, and in particular, no reports from the most vulnerable groups. This study aimed to examine the acute mental health impacts of the COVID-19 crisis as well as coping strategies employed by disadvantaged community members in North India.

**Methods:**

We used an intersectional lens for this qualitative study set in rural Tehri Garwhal and urban Dehradun districts of Uttarakhand, India. In-depth interviews were conducted in May 2020 during lockdown, by phone and in person using purposive selection, with people with disabilities, people living in slums with psychosocial disabilities and widows (total *n* = 24). We used the framework method for analysis following steps of transcription and translation, familiarisation, coding, developing and then applying a framework, charting and then interpreting data.

**Findings:**

The participants with compounded disadvantage had almost no access to mobile phones, health messaging or health care and experienced extreme mental distress and despair, alongside hunger and loss of income. Under the realms of intrapersonal, interpersonal and social, six themes related to mental distress emerged: feeling overwhelmed and bewildered, feeling distressed and despairing, feeling socially isolated, increased events of othering and discrimination, and experiencing intersectional disadvantage. The six themes summarising coping strategies in the COVID-19 crisis were: finding sense and meaning, connecting with others, looking for positive ways forward, innovating with new practices, supporting others individually and collectively, and engaging with the natural world.

**Conclusions:**

People intersectionally disadvantaged by their social identity experienced high levels of mental distress during the COVID-19 crisis, yet did not collapse, and instead described diverse and innovative strategies which enabled them to cope through the COVID-19 lockdown. This study illustrates that research using an intersectional lens is valuable to design equitable policy such as the need for access to digital resources, and that disaggregated data is needed to address social inequities at the intersection of poverty, disability, caste, religious discrimination and gender inherent in the COVID-19 pandemic in India.

**Supplementary Information:**

The online version contains supplementary material available at 10.1186/s12939-020-01345-7.

## Introduction

The COVID-19 virus has had huge impacts on health because of the dramatic social restrictions introduced to limit spread of infection, as well as the infection itself. The mental health effects of the COVID-19 crisis (defined as the pandemic and the associated control measures) are significant with millions of people in India and globally experiencing increased distress, anxiety, hopelessness and depression [1–3]. It is important to examine qualitatively because mental health is closely linked with physical health, behaviour and economic productivity which gives it importance beyond its intrinsic value [1, 4]. Similarly, examining attributes that support resilience, which can be understood as thriving and overcoming through stress or adversity [4], is key to understanding the most effective ways to support communities through and beyond the current COVID-19 crisis [5, 6].

In India the imposition of a series of strictly enforced lockdowns from March 23, 2020 halted most economic and social activity except some essential services. This had immediate flow-on effects such as widespread food insecurity, massive loss of employment, vast movements of migrant labourers and significantly reduced access to essential health services with these impacts increasing over the ensuing weeks [7, 8]. Around 40 million migrant workers lost employment and face significant food insecurity in the short or medium term [7, 9] and at the time of writing (November 2020), nearly 9 million people in India had been infected by COVID-19, with over 130,000 associated deaths [10].

Types of mental distress that increased during the COVID-19 crisis include increased stress, anxiety and tension particularly among health personnel working with COVID infected patients [1, 3, 9, 11]. Other groups at higher risk of worsening mental health include people with pre-existing mental health problems, and other disadvantaged groups including those who are poor, have less social support, are women and are experiencing violence [1, 2, 11].

Research priorities for global mental health in this pandemic include documenting the mental health effects of the COVID-19 crisis, with a descriptions of how these mental health consequences can be mitigated in disadvantaged groups as a notable research gap [12, 13]. In addition, there are urgent calls for research to focus on disparity and intersectionality, and to identify strategies that support resilience [7, 12, 14–16]. Holmes et al.; Wang et al. and Polizzi et al. [13, 17, 18] while there are a growing number of qualitative studies that examine the impacts of COVID-19 on mental health, most of these are set in high income countries or focus only on negative impacts [19–22] and no studies describe coping strategies to COVID-19 alongside description of the needs with a low income setting [19–22]. This focus is required in order to inform development of relevant and effective psychosocial interventions that can increase equitable mental health outcomes ahead [12].

The aim of this study was to examine both the acute mental distress linked to the COVID-19 crisis as well as practices that increased coping and resilience for disadvantaged community members in urban and remote rural areas of Uttarakhand state, North India.

## Methods

### Conceptual framework

Our focus in this study was to examine and represent the experiences of people who are systematically disadvantaged in India by using an intersectional lens [23, 24] which recognises that human identity is shaped by the interaction of different social and political identities which promote unique structural inequalities. We were keen to avoid a silo approach to tackling single-axis vulnerability and rather, to gather data that could portray a nuanced understanding of how disability, place of residence, and other social disadvantage can lead to impacts that are both positive and negative [23, 24]. We identified three key community groups: widows who are disadvantaged due to their gender, their risk of lower socioeconomic status and the stigmatising influences of widowhood [25, 26]. People with psychosocial disability (PPSD) residing in informal urban communities are disadvantaged by low socioeconomic status, the negative public perceptions and reduced access to services linked to the area where they live, and their stigmatising experiences of exclusion [27, 28] and thirdly, people with disabilities in rural areas who are disadvantaged by lack of access to services related to where they live, and by systemic structural exclusion with multiple barriers to participation [29, 30]. In describing the mental distress and coping strategies used by participants we aimed to consider the impacts of intersecting axes of disadvantage using an intersectional lens [31].

### Setting

This qualitative study is set in the districts of Dehradun and Nae Tehri in Uttarakhand state in North India during the time of lockdown. A stay-home order was in place and all economic activities were restricted except some related to health and food supplies and even public transport (the way poor and health-care workers reach health facilities) was discontinued [7, 32]. Mean measures of socio-economic status in Uttarakhand are lower than the Indian mean with greater poverty among people in remote regions. The rural Jaunpur block, Nae Tehri has few people and many steep hills with primary income generated by subsistence agriculture on terraced hillsides. Informal settlements in urban Dehradun are densely populated with temporary housing, and water taps and sanitation outside of homes. Both locations operate under a dominant patriarchy that favours men and disadvantages women [33–35]. While the National Mental health programme (NMHP) was sanctioned in 2016 across Uttarakhand, there are fewer than 10 Government psychiatrists for this state of 10 million people and none in Nae Tehri district. The authors are participant researchers: KM is a New Zealander who has worked and lived in Uttarakhand for 11 years. MR is of Scheduled Tribe identity from Uttarakhand, and along with KM, is employed by Herbertpur Christian Hospital. SP works as a psychiatrist in India and has lived experience of visual disability, while NG is an Australian who has worked and researched for over 15 years in Uttarakhand and has lived experience of disability. An overview of the study areas is shown in Table 1.

**Table 1:** Socio-demographic profile of the two study districts with state and national comparison data [36–38]

Indicator	India national	Uttarakhand state	Tehri Gharwal district	Dehradun district
Total population	1200 million	10.1 million	618,931	1,696,694
% population rural	72.2	69.5	88.8	44.5
% Households with access to improved sanitation (toilet)	44.9	64.5	65.8	75.6
% Prevalence of disability	2.1	1.5	1.2	1.7
% Prevalence of psychosocial disability	15.1	14.15	NA	NA
% Prevalence of widowhood	4.6	4.6	NA	NA
% Population residing in informal settlements	5.5	5.0	NA	NA

### Data collection and formats

Data was collected by team members from three non-profit organisations who were members of the Uttarakhand Community Health Global network cluster in May 2020 during the period of complete COVID-19 lockdown in India. Trained and experienced staff collected qualitative in-depth interviews with participants following a semi-structured interview guide which was developed by KM, NG and MR building from other interview guides and surveys we have used to assess experiences of barriers to participation, inclusion and exclusion for people with mental health problems and disability [30, 39]. The key domains of the interview guide included firstly, examining ways that the COVID crisis influenced day to day life and access to food, services and income, examining mental health impacts of the crisis,, and thirdly, probing practices and strategies employed by participants to improve their wellbeing, paying attention to ways that respondents long-standing intersectional axes of disadvantage interacted with the crisis to exacerbate or strengthen their responses. Interviews were undertaken by telephone or face to face where possible due to lockdown constraints.

### Participants

Participants were selected purposively by the participating community coordinator for each non-profit organisation participating, who resided in the community. Coordinators had long-standing relationships and frequent contact with people in the communities during the COVID crisis and were requested to recruit participants meeting inclusion criteria: people who had lived in the same community for the duration of the COVID crisis, who demonstrated a range of coping responses to the COVID crisis, who were socio-economically disadvantaged and who had at least and at least one further form of social disadvantage (for example, impacted by widowhood and/or disability and/or psychosocial disability). Some experienced disadvantage on multiple axes, such as one woman participant who was illiterate, a rural resident, a widow and from a disadvantaged caste. The only exclusion was people under that age of 18 years. People with visual, locomotor and intellectual disabilities were represented.. A total of 26 people were invited to join the study, and of these 24 gave written consent and 20 participants participated in the in-depth interviews. Four of the participants were too disabled to be able to respond in interviews themselves, and so we interviewed their caregivers. Demographics of the participants are outlined in Table 2 below.

**Table 2:** Participants in data collection

Characteristics	People with disability (*n* = 8)	People with psycho-social disability (*n* = 8)	Widows (*n* = 8)	Total (*n* = 24 participants)
Age (mean)	47	32	43	41
Education level (mean years of schooling completed)	7	8	1	6
Number of members in the household (mean)	5	5	3	4.5
Sex				
Men	5	3	0	8/24
Women	3	5	8	16/24
Main Activity (n)				
Employed full time	0	1	2	3/24
Self-Employed (farming)	8	1	6	15/24
Daily wage Earner	0	4	0	4/24
Looking after home	0	1	0	1/24
Student	0	1	0	1/24
Religion (n)				
Hindu	8	5	8	21/24
Muslim	0	3	0	3/12
Christian	0	0	0	0
Sikh	0	0	0	0
Caste (n)				
General	0	3	0	3/24
ST	0	0	0	0
SC	1	2	3	6/24
OBC	7	3	5	15/24
Marital Status (n) (Total in [%])				
Married	6	5	0	11/24
Widowed	2	0	8	10/24
Separated/ divorced	0	1	0	1/24
Never Married	0	2	0	2/24
Region (n) (Total in [%])				
Rural	8	0	8	16/24
Urban	0	8	0	8/24

### Data analysis

Interview recordings were translated and transcribed verbatim. We opted to use the framework analysis [40], for the following reasons: firstly, it is particularly good for data from interviews because it allows comparisons between and within cases, secondly, because it is well-suited for use by multi-disciplinary teams such as ours with public health, social science and psychiatric disciplines represented, and thirdly, because it provides a systematic model for managing and mapping the data which was important for a researcher team who were unable to meet in person due to the COVID crisis [41]. We followed the seven steps of framework analysis i.e. transcription, familiarisation, coding, developing and then applying a framework, charting and then interpreting data [41]. MR and KM read and re-read the transcripts to familiarise themselves and then and coded the first three interviews and then compared and contrasted coding to develop a working analytical framework. KM then coded remining 21 interviews, continuing to adapt the framework to reflect emerging themes. The analytical framework with 216 codes was then displayed in a matrix using Microsoft Excel (2016), and findings were compared and contrasted to develop 19 categories, with a focus on how intersectional social identity played out in these categories, and subsequent themes. These were reviewed and discussed with NG and MR and further modified to better reflect observed patterns, and then again analysed going back and forth between the transcripts, codes, categories and themes until six themes each under the headings of COVID-19 contributions to mental distress and coping were agreed upon in the final step of data interpretation. Within each of these we paid attention to intersectional influences on the theme and described experiences and practices, and particularly unpacked these in the theme titled ‘Intersecting disadvantage’. The source of verbatim quotes is noted with this convention unless this detail could compromise anonymity: (Sex, Age, group/s they belonged to Widow, Migrant Labourer, Person with Disability). A table in the Supplemental material shows the grouping of categories to themes, and the intersectional considerations within each theme.

### Ethical considerations

To ensure the study addressed ethical considerations, all participants were provided with a package of food rations, and all were offered ongoing psychosocial support with counselling through the non-profit organisations we worked with. All participants were contacted within 3 weeks of completion of the interview to ask if further psychosocial or other support was required. Several participating families were referred for further support with food rations. All participants gave informed consent. To elicit consent from study participants with mental health problems which impacted their capacity for consent, or those with cognitive disability, we took written consent from their primary caregiver as well as the participants.

We conducted face to face interviews in a separate room or space to ensure privacy. To maximise privacy for telephone interviews for people living in small one-roomed houses, we suggested participants sit on a roof, or outside away from other household members, and held interviews at a time of day chosen by participants where this was possible. The study was approved by the Emmanuel Hospital Association Institutional Ethics Committee in April 2020 and assigned protocol number 229.

## Findings

Findings of this study are summarised under the meta-themes and realms where they were evident in Table 3.

**Table 3:** An overview of key themes of the study and the realms where they occurred

Mental distress and coping level	Mental distress linked to COVID19	Coping with COVID19
Intra-personal	Overwhelmed and bewildered	Finding sense and meaning
	Stuck locally, connected globally	Looking for positive ways forward
	Distressed and despairing	Innovating with new practices
Interpersonal relationships	Feeling socially isolated	Seeking psychosocial support by connecting with others
Social and environmental	Intersecting disadvantage	Supporting others individually and collectively
	Othering, discounting and discriminating	Engaging with the natural world

### Six themes related to mental distress during the COVID crisis

The mental health impacts experienced by participants in this study are summarised under six themes.

#### Overwhelmed and bewildered

Participants described the pandemic as overwhelming and novel, describing many unknowns. People with lower literacy (slum residents and people with cognitive and physical disabilities), and women heads of households (without mobile phones) narrated that they had repeatedly requested neighbours or relatives to explain news updates and new rulings in simple terms, as well as seeking instructions for control measures they should follow (for example how to go about life in lockdown). Participants with multiple social disadvantages described limited their ability to respond appropriately and left them feeling incapable with this new threat. F or example, a widow who was not literate stated ‘I am just a woman and not educated so I how can I know what to do? I know nothing about this new situation.’

Participants described fear about widespread systems failure, lack of leadership, failing health systems and also described the capricious and unpredictable aspects of the crisis such as the wild cattle in Jaunpur block of Tehri Garwhal, who had gained boldness with reduced traffic to destroy many villagers’ crops. The pervasiveness and unprecedented impacts of COVID-19 was described as eclipsing all other difficulties and diseases as described below:

*This is a very dangerous disease. I have never heard about it in my life and now we have forgotten about all other diseases. We can only recall corona now. () Now there are no more diseases like dengue, malaria. There used to be so many diseases in summer, but now we have only this one disease and all the other fevers have vanished.*

**M, 42, Carer of person with disability**

#### Stuck locally, connected globally

The experience of the pandemic was simultaneously very local and very global. Participants described restrictions in due to stay-at-home orders requiring them to be geographically based in their small house, urban settlement or village for many weeks, meaning they were physically more constrained than ever before. At the same time, participants described seeing more global media than ever before, and also described knowledge via relatives, of far-off places such as the overflowing hospitals and increasing rates of COVID-19 in Mumbai. Yet the scarcity of local cases of COVID led to rural participants describing COVID-19 asalmost fictional, as illustrated below:

*(When I heard about corona virus) I thought what can actually happen to us out here? We don’t feel like much would happen as we live in a remote place but we heard about it happening in the plains, like from my brother who is stuck down there.*

**F, 37y, Widow and carer of person with disability**

#### Distressed and despairing

Increased fear, anxiety and mental distress due to the repeated lockdowns was a feature for all participants. Mental distress about future food security was prominent for those with high material poverty and their narratives focused on the need for essentials rations and the future for their children. This led to a sense of impending doom as illustrated below:

*I was really tensed and scared, every time I was thinking about what we would eat next and how is this all actually happening. I also was thinking that the world would end soon, and the only question was where we would die. At home or in the jungle.*

**F, 37y, Carer of person with disability**

Mental distress was added to for people with psychosocial disability, as well as those with chronic conditions like hypertension as they were running out of medicines and not able to access medicines or attend health services due to lack of public transport in lockdown. They didn’t know how this would affect their pre-existing health problems. Fear of COVID-19 infection was a greater concern for those in informal urban settlements especially for those with family members in distant cities, as well as those with least education and few knowledge resources in their households (e.g. illiterate widows).

#### Feeling socially isolated

The reduced social contact required by the lockdown was difficult, particularly for women heads of households, who described their usual forms of coping was to visit family and friends nearby. Some felt that using a phone was a substitute for social meetings while others felt it was not, or they did not have funds to pay for calls, and most described increased feelings of isolation. Participants from low-quality housing areas of Dehradun also described the negative social outcomes for children not being able to go outside and play and that there were increases in arguments and unhappiness for the whole family, particularly in the crowded single room households .

*It doesn’t feel good to keep ourselves at home only. We cannot roam around at all as it might lead to infections and this situation has* apne paraye sab door ho rakhe hain *(kept us from our own folks).*

**F, 55y, person with disability**

#### Intersecting disadvantage

Greater impacts on food security and despair were described by participants with intersectional disadvantage, than those with a single disadvantage. The role of loss of income for people working as daily-wage labourers was most prominent among people in informal urban settlements as well as rural residents with disabilities and without land. Parents described concerns about their children’s studies especially if they didn’t have a smart phone and described feeling further disadvantage if they couldn’t access schoolwork like children who were doing ‘classes by Whats app’. Illiterate women participants also underlined their lack of access to information about COVID For some women with multiple forms of disadvantage, the lockdown meant there was almost nothing to eat. One widowed, illiterate OBC woman with a child with a disability described:

*We are eating a lot of fried rice cakes and drinking tea but we are also hungry ( ) In the lockdown I went to the market and I had a single rupee, and there I found a man from our village. He was buying things from a shop. I asked him to help me with Rs200 and I purchased and took some more items on credit.*

**F, 28y, Widow and carer of person with disability**

#### Othering, discounting, discriminating

There were frequent references to ‘others’ referring to people possibly with COVID-19 infection, returning migrants, labourers from ‘outside ‘(such as Kashmir). Increased othering and negative judgement towards other religious groups was evident , with participants in Dehradun (with equal numbers of Muslim and Hindu) alluding to the risk of infection from a religiously linked group who had a cluster of infections early in the Indian COVID epidemic. As well as descriptions of increased mistrust and negative judgement towards people from the ‘other’ religious group. A woman describes this:

*People from the Muslim community are all not following the rules ( ) and there is a change in our relations since COVID ( ). Earlier the women in the neighbourhood used to say ‘tum meri dharam ki behen ho’ (that you are my sister in faith) but now they are ready to run bulldozers over our homes. They threaten us over everything, and they are full of hatred.*

**F, 41y, PPSD**

Within households, people with greater autonomy discounted the impacts for other family members. For example, male participants described how the crisis affected them the most, seeming to discount affects on family members who were women or disabled. A father of a child with disability illustrates this below:

*(My wife and grandmother) do not feel much about COVID as they stay at home and don’t have much to do with these things (*referring to COVID and national news)*. They are just busy with their work and for them it is all the same, they eat food and work in the fields. ( ) But I am affected as I am the one in this house who goes out.*

**M, 31y, Carer of person with disability**

### Six themes related to coping with the COVID crisis

Our findings identified six key strategies for coping and resilience occurred in the intra-personal, interpersonal and wider realms.

#### Making sense and meaning

Participants described ways they found meaning in the COVID-19 crisis. Firstly, through the practices and beliefs of their religion where some described solace in knowing all events are in the hands of *Uparwala* (the One Above) or described following more frequent devotional practices. Secondly, participants described that the COVID crisis as more bearable than a singular family crisis as it affected rich and poor alike, finding a sense of camaraderie with all of humanity impacted by this global pandemic. For example, a wheelchair user described how years back, he and his family had found forms of income despite his spinal injury which reassured them that they could also navigate the emerging stresses of the COVID crisis.

*“Just as we got through hardship after my back injury before, we will get through again. And in current pandemic all are suffering: this fight is not alone and others out there also going through the same thing.”*

***M,40y,*** **person with disability**

Thirdly, participants described reassurance in closely complying with guidelines on handwashing and social distancing. Control measures for some were talismanic and rigid as a young man illustrates:

*(To cope with our fear) I put on a mask, and wear gloves and maintain social distancing. My mother gets scared and says we should not venture out of the house. I tell her that we should maintain two metres distance between each other instead of one. My father is staying home and also my brother and even wear their masks inside our house. Even while serving food we wear gloves, ( ) take our food and each sit in different corners of the room.”*

**F, 20y, PPSD**

#### Looking for positive ways forward

Actions for self-care, positive thinking and finding benefits emerged as key strategies to adapt positively. For example, people described choosing not to watch negative or anxiety provoking news channels or choosing not to dwell on the difficult times as described below.

*These things [referring to financial hardship] keep playing on my mind but I distract myself otherwise it will affect me more. If we keep thinking about it, it will affect me and my family. It’s better to not think too much, it will not change anything.*

**M, 48y, PPSD**

Participants described the value of finding benefits from the lockdown, such as greater time to play games with children, a new healthy habit (like a fitness programme) and more family time together. Participants even valued self-care practices over finances, for example a woman who continued with her newspaper subscription, although they didn’t even have tealeaves at home:

*“Every day I try to find something hopeful in the paper about the end of this lockdown situation ( ) Then my neighbours also read the same newspaper, and although my kids ask me to stop wasting money ( ), I tell them we can afford it.”*

**F, 42y, PPSD**

#### Seeking psychosocial support by connecting with near and far

Central in coping was communication with family and friends, with nearly all participants described increased frequency of contact and longer conversations More than one participant described that ‘I would have died without my phone”. as quoted below:

*If there were no mobile phones then only God knows how people would have kept themselves going and entertained too. And those away from their homes in these times would have died by now from missing their own people (Agar kisi ko mummy ya papa ki yaad aati toh vo toh sir patak patak ke hi mar jata)*

**F, 18y, PPSD**

#### Supporting others individually and collectively

A key coping strategy was helping others, with participants describing spending more time than usual with family members in need, like an older person or child with disability, as well as helping neighbours as described by a widow in the quote below:

*“There is a lady who lives far away. She is alone. I keep sending her food. We also send ration to her. She is just like our grandmother. We want her to be well. Others in her neighbourhood also help her, they give her cooked meal, and it’s the least we can do for her right now”.*

**F, 27y, Widow**

Participants also described how they had taken collective action, for example, in one rural village, residents set up a roster to provide cooked meals to a migrant labourer from Kashmir who was marooned in their village. Rural participants described how in their village they had agreed to all contribute to travel for city workers to help them return to their rural homes and had also collaboratively agreed on where to house them and how to ensure they followed quarantine with a supply of food.

#### Engaging with the natural world

Renewing connection to natural resources was a key strategy used by participants who described natural resources such as pollution-free blue skies as well as time in fields as making their minds lighter (‘*mannhalke ho jatehai’*). This engagement was context specific so that in Dehradun settlements, participants described children finding solace with pet fledgling birds while adults looked out from their rooftop at dawn or dusk. Those in rural areas described spending more time in the fields and with their pastoral animals as a widow described below:

*(To get peace of mind my children) are going out to work in the fields and others are going to the jungle. When you take oats grazing you don’t remember that corona is happening.*

**F, 27y, Widow**

#### Innovating with new practices 

Participants described starting new hobbies such as ainting or writing poetry and working harder than usual to distract themselves from their anxieties. One young woman described that she and her other Class 12 friends had decided together that each year all of society should go into lockdown for a month for mental and physical wellbeing.

## Discussion

To the best of our knowledge, this is the first study using primary data that has qualitatively examined mental distress and coping responses to COVID-19 in South Asia with an intersectional lens. The impacts of COVID-19 on mental distress align with quantitative studies that also show that there are far-reaching mental health consequences such as reduced sense of control, social isolation, increased social 'othering' and discrimination. [1, 2, 42]. While this study recognises the systematic effects of intersectional social disadvantage, we also recognise that responses to stress are mediated at the individual level where stress is primarily experienced when demands exceed resources (the ability to cope with and mediate stress) as proposed by Folkman’s transactional model of stress and coping [43]. Thus, the interpretation of a stressful event by an individual can be more important than the perceived dimensions of the event itself. Some participants in this study were significantly impacted by loss of income and reduced food security due to the COVID-19 crisis (linked to their place of residence, disability, gender or educational status) yet did not consider their internal resources and hope overwhelmed, and thus employed coping strategies described. Other participants appeared to have adequate resources such as food, social capital and shelter, yet experienced despair. The contribution of the transactional theory of stress and coping can thus be evident at an individual level where all participants were actively engaged in a circular process of primary appraisal, cognitive and behavioural coping efforts, adaptation and re-appraisal, and at the same time, our findings show that intersectional identity and systematic disadvantage also interacts at multiple locations of the process of balancing coping and stress .

Compounding disadvantage related to intersectional identity such as low literacy, female gender, disability, landlessness, dwelling in poor urban housing areas and widowhood led to increased and significant mental distress for most participants. Our findings suggest people already disadvantaged by their social identity and structural exclusions are more likely to experience further social exclusion, greater health needs, reduced access to care and ultimately, worse outcomes in the COVID-19 crisis [7, 32, 44], which has acted as an inequality amplifier [16, 27, 45]. Women in our study particularly reported bewilderment and low knowledge about COVID-19, and other intersectional studies of disasters have shown that women are more likely than men to be disadvantaged due to their reduced access to the protective attributes such as literacy, education, paid employment and political decision-making which are critical resilience factors in disaster [4, 15]. People in informal urban settlements with already limited access to resources can experience extortionate costs to access water or toilets and are often not represented by formal or informal governance structures [27]. Research that does not seek to include and represent the perspectives of those with compounded disadvantage, fuels inequalities in policy [16, 27, 45]. Current reporting of COVID-19 in India focuses on the demographics of age and state, with almost no publicly available COVID-19 data disaggregated by social determinants such as disability, religion, caste and rurality (personal communication with others researching disparities in India). This study joins the call by others for research and data that uses an intersectional lens and disaggregated data in order to address the social inequities at the intersection of poverty, disability, caste and religious discrimination and gender [7, 12, 23, 45, 46] and further calls for increased attention to the assets and resources employed by individuals [43] and communities to cope and be resilient in adversity such as the COVID-19 crisis [47].

This study additionally underlines the importance of qualitative research methods which can provide greater depth and nuance in findings that acknowledge the complexities of life-course adversity and coping during infectious disease outbreaks [16, 48] and thus guide development of context-relevant responses. Our findings demonstrate that people use creative psychosocial practices to mitigate the mental distress caused by COVID-19, a finding shared by a similar study after the Asian tsunami in 2004 which documented coping strategies such as finding meaning, benefit finding, the benefits of religious belief and practice and community cohesion and support [49] and supported by transactional theory of coping and stress [43]. Interventions can build on practices by individuals or communities such as nature-based coping strategies such as engaging with animals and or community gardens [50]; can support thinking positively by practices of gratitude and benefit finding [18], and religious leaders (already identified as a community resource), who offer psychosocial support with their congregations. Currently these potentially supportive resources are not identified or implemented partly because we do not have systems for identifying, representing and supporting these approaches in communities [44, 48, 49].

Increased ‘othering’ and discrimination (by other religious groups or towards returning migrants) was amplified by fake news and the storm of social media which many studies have shown in the COVID-19 crisis [27, 42]. Our findings of reduced perceived social support and trust in others is described in other infectious disease outbreaks [27, 44, 51]. Stigma is tightly linked with exclusion and injustice, and is an independent health determinant [52]. The loss of trust between different community groups risks increase in social isolation and loss of trust, both important protective attributes for psychological wellbeing [49, 53]. Othering of health workers, people with COVID-19 infection and migrants, and increases to pre-existing prejudices related to caste, ethnicity and religion, which is a significant feature of this pandemic [12, 16, 44]. Where the individual possesses a number of these identities then discrimination has been compounded during the pandemic. Public policy with specific strategies such as affirmative action, public education and suppression of fake news and misinformation in order to increase social inclusion is urgently needed.

Our study found people without a phone (nearly all were women head of households) described greater bewilderment, reduced awareness about how to manage mental distress and felt further disadvantaged as their children couldn’t access educational resources compared to those with access to media and a mobile phone. Technology has been valuable for many during COVID because of its’ applications which can enable our social, educational and occupational lives to continue, and because it can facilitate social participation, inclusion and mental health [44, 54–56]. Our findings align with these other examples which underline the importance of the mobile phone and other digital technologies in coping with COVID-19 as a key structural support to mental health, communication, education and access to information [54, 56]. While India’s central government, through the Department for the Empowerment of People with Disability, has issued official notices to states to implement their obligations, there are ongoing issues of digital disparities for people with disability such as the low accessibility of the Aarogya Setu App for the visually and hearing impaired. To increase information and promote wellbeing in the face of a disaster, there is an urgent need to increase access to digital technology for the most disadvantaged groups. Mobile phone technology will be increasingly important in India where there were over 1000 million mobile users in 2019 [57] however, interventions relying on mobile phone technology have limitations as the most vulnerable may not have access to a smart-phone and adequate data allowance, or a disaster may cut phone signals and electricity supplies. Although, mobile phones can play an important role in messaging and wellbeing, parallel strategies to ensure those most disadvantaged have access to information are also required.

Participants with chronic conditions including psychosocial disability could not access care or essential medicines due to the COVID-19 crisis which underlines this as an unfolding economic, social, humanitarian and health disaster in which states are obliged to ensure the protection and safety of people with disability [58, 59] under Article 11 of the Convention on the Rights of Persons with Disabilities (CRPD) and section 8 of the Rights of Persons with Disabilities Act, 2016 (India) [60, 61]. The intersectional disadvantages for people with disabilities in our study were evident in their reduced access to social protection and welfare such as rations due to reduce mobility and public messaging. These barriers to protective supports lead to further inequities in outcomes [44]. To respond to the unfair and preventable inequalities in the COVID-19 crisis policy makers need to use a public health framework to respond to COVID-19 in India [7] proposes that comprehensive Government responses focus on the three areas of policy: social protection (with a focus on those most vulnerable), continued provision of essential health services and specific responses control of the COVID-19 epidemic.

It is important to ensure access to care for those with pre-existing health conditions particularly for disadvantaged groups who experience intersecting and additive disadvantages, and this should include diverse and far-thinking interventions such as supporting community participation in governance of informal settlements [27], ongoing provision of public transport (to access services) as well as ensuring there are accessible and operating health services [7, 32]. Further, accessible social protection through welfare systems that address the needs of people with multiple and intersectional disadvantage [7] is urgently required. In the longer term, use of participatory and inclusive design to deliver equitable system responses in ‘peace’ time, increases the likelihood of equitable policy and outcomes in a national and global disaster [7, 15, 27].

### Methodological considerations

There are limitations to this study: as data collectors were women, male participants might have spoken less freely; use of phone interviews for some may have also reduced open communication As participants were identified as known clients to recognised non-profit health organisations, they are likely to have had increased social and knowledge resources available than others in the wider community (meaning negative influences may be greater and coping strategies more limited) than reported here, and may also mean that responses were coloured by social desirability bias. However, to mitigate this we used formats and probing questions that encouraged open and honest responses and triangulated findings with four team members of Herbertpur Christian hospital working in the community who reviewed and responded to findings in the light of their community-based field work to increase the study’s credibility. Additionally, three further strategies promote the trustworthiness of this study: transferability, dependability and confirmability. We maximised transferability by providing in-depth contextual detail and believe many settings in India would share characteristics of the study site, whiledetailed descriptions of context, methods and analysis enhanced dependability and confirmability.

## Conclusions

Analysis of mental health experiences and coping strategies of intersectionally disadvantaged groups in the COVID-19 crisis underlines how far-reaching the crisis is on all aspects of human life, and points to the importance of including people with lived experience; and designing and implementing equitable interventions that build on existing community resources. Attention to social health determinants and the principles of universal design in messaging, technology, social protection and health services in the COVID-19 pandemic is requisite to increase equity in outcomes for people already disadvantaged by the political economy of health.

## Supplementary Information

**Additional file 1: Table 4.** Framework analysis of categories highlighting intersectional aspects of each theme identified, under the meta-domains of Impacts of and Coping with the COVID-19 crisis.

## Data Availability

The datasets used and/or analysed during the current study are available from the corresponding author on reasonable request.

## Abbreviations

F: Female
M: Male
PPSD: Person with psychosocial disability
PWD: Person with disability
