# Supplementary material for: “We’ve got through hard times before: acute mental distress and coping among disadvantaged groups during COVID-19 lockdown in North India - a qualitative study”
Source: Int J Equity Health. 2020 Dec 17;19:224. doi: 10.1186/s12939-020-01345-7 (PMC7745174; doi:10.1186/s12939-020-01345-7)
Supplement: Supplementary file 1 — Additional file 1: Table 4. Framework analysis of categories highlighting intersectional aspects of each theme identified, under the meta-domains of Impacts of and Coping with the COVID-19 crisis. [file 12939_2020_1345_MOESM1_ESM.docx]

Supplementary material

**Table 4.** Framework analysis of categories highlighting intersectional aspects of each theme identified, under the meta-domains of Impacts of and Coping with the COVID-19 crisis

| **Meta-theme** |  | **Themes** | **Categories** | | | | **Intersectional considerations** |
| --- | --- | --- | --- | --- | --- | --- | --- |
| IMPACTS | 1 | **Overwhelmed and bewildered** | Losing jobs increases anxiety for future | Students worried about their future | Changing family relationships | | Those without phones or access to media (i.e. women, people with disabilities and young people) most overwhelmed – describing reliance on others for information about how to stay safe, and follow new rules. |
|  | 2 | **Stuck locally, connected globally** | Reduced freedom of movement | Sense of widespread systems failure linked to globalization | Rural & urban different location, different experiences | | This theme particularly underlined differential impacts based on geographical location (rural areas had greater freedom of movement and went to fields) but was cut off from information. Those with smartphones or access to TV or radio had a stronger sense of global connection than the poorest that did not have access to any media. |
|  | 3 | **Distress and despairing** | Mental distress exacerbated by anxiety re: COVID | Uncertainty regarding future increases distress | Unable to access health services, medicines or other supports | | People with multiple disadvantage most anxious and distressed – especially women, those with low agency (with disability or without income), and those with COVID linked loss of household income. |
|  | 4 | **Feeling socially isolated** | Wanting face to face interaction | Reduced social and religious gatherings | Wanting information on COVID and social support | Reduced freedom of movement for women especially | These categories highlight increased experience of social isolation without usual social interactions. Women, people with disabilities and the poorest i.e. those without social/ media access particularly isolated without usual religious or social gatherings, while men continued to move beyond house with associated social benefits. |
|  | 5 | **Intersecting disadvantage** | Finances and food security dominant concerns | Vulnerabilities additive for marginalised | Unequal support- some getting better other getting worse | | Those with multiple disadvantage (residence in poor urban or rural area, widowhood, disability or household member with disability, low literacy, low or no income) impacted on every sphere to hugely increase impacts. |
|  | 6 | **Othering, discounting, and discriminating** | Blame and stigma related to COVID | Discounting women and PWD | | | Discounting of the value or impacts of COVID crisis faced by women, widows or person with disabilities in particular, creates a sense of othering and also adds to other structural disadvantages experienced by already excluded groups. |
|  |  |  |  |  |  |  |  |
| COPING | 1 | **Making sense and meaning** | Complying to safety rules | Finding ways to practice religious devotion | Recognising that all near and far are impacted by COVID | | This theme seemed to have little nuance by intersectional disadvantage – people with multiple disadvantages showed resistance to being overwhelmed by making meaning from the crisis. |
|  | 2 | **Looking for positive ways forward** | Finding benefits in difficult situation | Positive thinking and trusting authorities act in public interest | Taking care of self | | Those with multiple intersectional disadvantages most trusting of authority, while other categories spread evenly across intersectional identities. |
|  | 3 | **Seeking psychosocial support by connecting with near and far** | Communicating via phone or Smartphone a lifeline | | | | People with access to any phone and with funds to pay for connectivity found this critical support. However widows and people with disabilities particularly described feeling of increased isolation due to lack of access to phone. |
|  | 4 | **Supporting others individually and collectively** | Helping others in neighbourhood | Spending quality time with family | Increased companionship and time with neighbours | | Despite disadvantage, multiple participants described COVID crisis as opportunity to spend quality time with family and building better family and neighbourhood relationships. Widows and people with disabilities more likely to receive than offer support. |
|  | 5 | **Engaging with the natural world** | Finding peace in nature | Rural residents increase value of time in field and with animals | Urban residents increase value of outside, even rooftop, terrace and birds | | Stay-at-home order limited usual movement and new creative ways to engage with natural world. This was practiced differently in rural and urban areas, but both conveyed sense of engaging with what was there. |
|  | 6 | **Innovating with new practices** | Distraction and keeping busy as strategy for wellbeing | Developing new practices of wellbeing: painting, yoga or reading etc. | | | Keeping oneself busy or distracted by new practices was a key practice but more limited among those with reduced access to technology or with low literacy. |
